# Reflections on the HUPO Human Proteome Project, the Flagship Project of the Human Proteome Organization, at 10 Years

**DOI:** 10.1016/j.mcpro.2021.100062

**Published:** 2021-02-26

**Authors:** Gilbert S. Omenn

**Affiliations:** University of Michigan Medical School, Departments of Computational Medicine & Bioinformatics, Internal Medicine, Human Genetics, and School of Public Health, Ann Arbor, Michigan, USA

**Keywords:** Human Proteome Project, Mass Spectrometry Data Interpretation Guidelines, neXtProt, missing proteins, functionally unannotated proteins, blueprint, HPP, Human Proteome Project, HUPO, Human Proteome Organization, MP, missing proteins according to neXtProt, MS, mass spectrometry, PRIDE, Proteomics Identification Database, PTM, posttranslationally modified, SRM, selected reaction monitoring, TPP, Trans-Proteomic Pipeline

## Abstract

We celebrate the 10th anniversary of the launch of the HUPO Human Proteome Project (HPP) and its major milestone of confident detection of at least one protein from each of 90% of the predicted protein-coding genes, based on the output of the entire proteomics community. The Human Genome Project reached a similar decadal milestone 20 years ago. The HPP has engaged proteomics teams around the world, strongly influenced data-sharing, enhanced quality assurance, and issued stringent guidelines for claims of detecting previously “missing proteins.” This invited perspective complements papers on “A High-Stringency Blueprint of the Human Proteome” and “The Human Proteome Reaches a Major Milestone” in special issues of *Nature Communications* and *Journal of Proteome Research*, respectively, released in conjunction with the October 2020 virtual HUPO Congress and its celebration of the 10th anniversary of the HUPO HPP.

## Highlights

• The global Human Proteome Project is the flagship activity of the HUPO.
• HPP Guidelines for MS Data have greatly enhanced confidence in proteomics data.
• The community has identified proteins from 90% of predicted protein-coding genes.
• A total of 1899 predicted proteins lack sufficient evidence of expression as of 2020.

## Graphical Abstract

**Figure:**
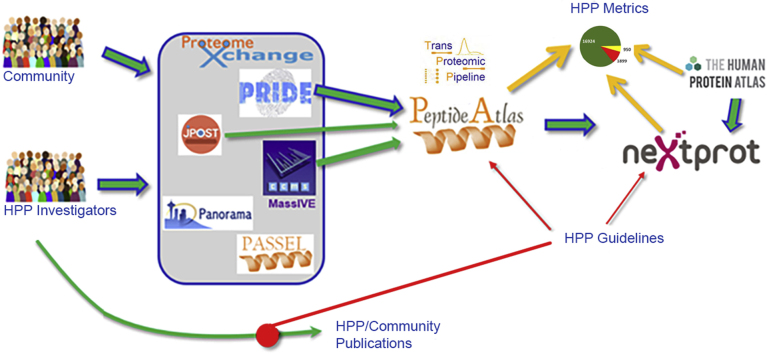

## In Brief

Starting from several organ-oriented projects, HUPO in 2010 launched the Human Proteome Project to identify and characterize the protein parts list and integrate proteomics into multiomics research. Key steps were partnerships with neXtProt, PRIDE, PeptideAtlas, Human Protein Atlas, and instrument makers; global engagement of researchers; creation of ProteomeXchange; adoption of HPP Guidelines for Interpretation of MS Data and SRMAtlas for proteotypic peptides; annual metrics of finding “missing proteins” and functionally annotating proteins; and initiatives for early career scientists.

During the lifetimes of my contemporaries, we have learned that triplet-nucleotide sequences of double-helical DNA carry the code of heredity, that normal human cells have 46 chromosomes, and that proteins carry out an amazing variety of structural, metabolic, catalytic, immune, regulatory, and signaling functions. The “central dogma of molecular biology” is that genetic sequence information in DNA is transcribed via heterogeneous nuclear RNA into mRNA messengers, which then program their translation on ribosomes to amino acid sequences of proteins. Remarkably, the protein sequence determines the folding and functions of the protein (1). Of course, the details are more complex. However, a crucial observation, not sufficiently appreciated in the world of genomics, is that direct study of proteins is essential for cell biology, biochemistry, physiology, and precision medicine. Predicting the dynamics over time of protein abundance, intracellular localization, transport, secretion, and intermolecular interactions of proteins—and their splice isoforms and posttranslationally modified (PTM) proteoforms—is not feasible from DNA or RNA studies. Moreover, protein transcription factors and RNA-binding proteins play crucial roles in regulating gene expression, and proteins are the targets of most modern drugs.

As the Human Genome Project (HGP) progressed, there were such major surprises as the observation that only 1.2% of the sequence was represented in protein-coding genes, revealing ignorance or uncertainties about the roles of the rest of the DNA. Estimates of the numbers of proteins ranged from 50,000 to 100,000 and higher; by the time the HGP sequences were released, the estimate had declined to 35,000. Now we know that there are about 20,000 protein-coding genes, but the number of functional proteins is a multiple, due to alternative splicing and many combinatorial posttranslational modifications. During recent years, the diversity of RNAs and their many functions in gene regulation also have been revealed.

My favorite relevant cartoon appeared in the *Times of London* just 5 days after the publication on February 15 and 16, 2001, of the landmark special issues of *Science* and *Nature* with the proposed sequences for about 90% of the Human Genome. The message was “Searching for the Real Stuff of Life!” It dramatically depicted a lively globular protein at center stage, with the DNA double helix unceremoniously being moved off-stage. Earlier, *Business Week* ran a story headlined “Biotech’s Next Holy Grail: Companies are Racing to Decipher the Protein Set”.

Indeed, proteomics got a major boost at that time, with advances in mass spectrometry and NMR recognized with the 2002 Nobel Prize in Chemistry to John Fenn, Koichi Tanaka, and Kurt Wuthrich. The Nobel announcement stated that “chemists can now rapidly and reliably identify what proteins a sample contains ... and how they function in the cells.” These are the twin goals for proteomics. There were investments by pharmaceutical, chemical, and instrument companies and the launch of new journals, notably *Molecular & Cellular Proteomics* and *Journal of Proteome Research.* A landmark *Nature* paper by Aebersold & Mann (2) provided a primer for the several types of MS instruments; they addressed detection and quantitation of protein binding partners and PTMs, presented integrated analyses of the *Falciparum* malaria parasite and its hosts and organellar biology of the nucleolus, and called for much deeper publication of databases.

## Formation of the Human Proteome Organization (HUPO) and Emergence of the Human Proteome Project (HPP)

An important development, stimulated by the prominent role of the Human Genome Organization (HUGO), was the convening of interested scientists in Virginia in 2001, organized by Samir Hanash, to create the Human Proteome Organization (HUPO) and plan its first World Congress of Proteomics in Versailles in Fall 2002 (3). Our mission was to mobilize scientists around the world across academic, industrial, and government sectors in proteomics research and development, to attract young scientists to this exciting new field, and to stimulate and coordinate scientific initiatives. Those multinational initiatives began with the Plasma (4), Liver (5), and Brain (6), then Kidney/Urine (7) and Cardiovascular (8) Proteome Projects alongside the Protein Standards Initiative coordinated by the European Bioinformatics Institute (9). A complementary development was the Human Protein Atlas (HPA) in Sweden (10), generating antibodies to identify and localize proteins in tissues and organelles with immunohistochemistry.

By 2008, there were discussions at the HUPO Congress in Amsterdam with funding agencies and many leading scientists about organizing a large consortial effort. There was strong support for HUPO to function as a convener and facilitator, not competing with investigators or academic institutions for national or international funding.

In September 2010, participants at the Sydney HUPO Congress endorsed the launch of the Human Proteome Project. I was sent to the studio of Australian National TV to be interviewed on this news-worthy event! The two major goals of the HPP were and remain: (a) building a “protein parts list” based on highly credible evidence of detection of expression of one or more gene products from each of the approximately 20,000 human protein-coding genes, and characterizing the functions of those proteins and their many proteoforms; and (b) making proteomics a widely deployed component of multiomics research in health and disease. The launching article about the HPP was published in *Molecular & Cellular Proteomics* in 2011 (11).

## Organization of the Human Proteome Project (HPP)

**Fig. 1:**
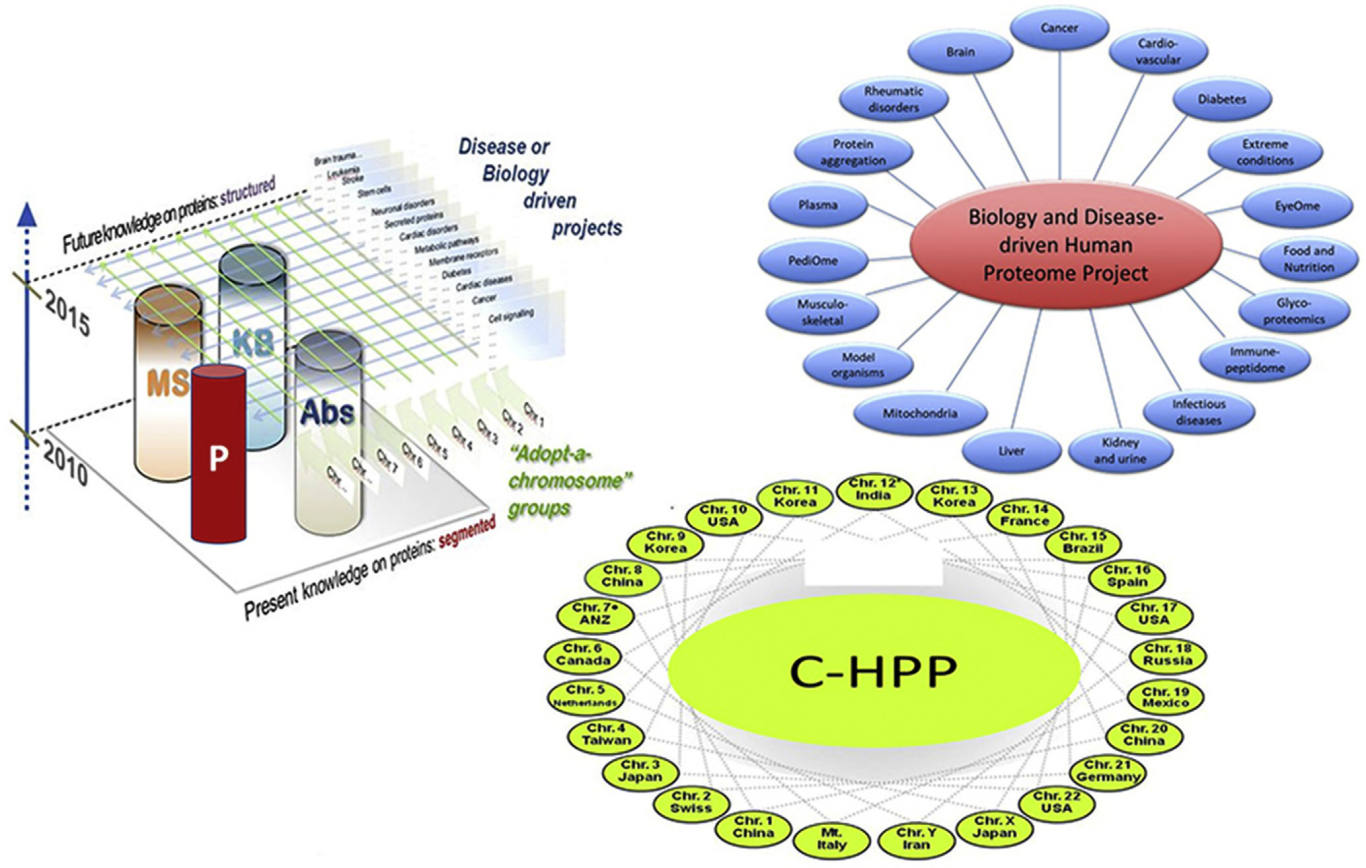
**Schema showing the matrix structure of the Human Proteome Project (HPP).** There are 25 chromosome-centric HPP teams corresponding to chromosomes 1–22, X, and Y plus mitochondria, with lead country shown. There are 19 Biology and Disease-driven HPP teams, and four Resource Pillars, of mass spectrometry, antibody profiling, knowledge base, and pathology. MP-50 and CP-50 refer to the C-HPP challenges to find 50 missing proteins per chromosome and generate functional annotations for 50 uncharacterized PE1 proteins. See text.

Led by Young-Ki Paik and William Hancock, and later Chris Overall and Lydie Lane, the chromosome-centric HPP (C-HPP) brought together teams focused on each of the 24 individual chromosomes and mitochondria. This strategy represented an analogy to the HGP and a division of labor, with opportunities for 25 teams of proteomics researchers in many nations or regions around the world (Fig. 1). We were well aware that functionally related proteins in metabolic or signaling pathways are often coded by genes on different chromosomes; however, we recognized a biological rationale from genes coexpressed in amplicons, families of homologous proteins from duplications, and cis-regulatory phenomena. The C-HPP initiated MP-50 and CP-50 challenges to detect 50 missing proteins per chromosome and functionally annotate 50 uncharacterized uPE1 proteins (see Fig. 1 and text below). The C-HPP formed a partnership with the *Journal of Proteome Research* to produce an annual special issue of articles from the HPP investigators and from the community at large. From 2013 through 2020, a total of 204 papers have appeared in these eight special issues.

Provided by Young-Ki Paik and Jin-Young Cho, adjusted by Aaron Bookvich.

Simultaneously, the biology and disease-driven HPP initiative (B/D-HPP), chaired by Ruedi Aebersold, and later by Jennifer van Eyk, Fernando Corrales, Ileana Cristea, and now Jennifer van Eyk, brought together the existing organ-based proteome projects and stimulated many additional teams. As expected, publications from these 19 groups (Fig. 1) have been spread over numerous journals reflecting the biological processes and clinical objectives. The most recent is the Human Immunopeptidome Proteome Project. The HPP Human Proteome Resource Library search in 2020 provided a broad catchment of publications in these fields (12). Special products from the B/D-HPP include the vast SRMAtlas (13) and bibliometric analyses of the most popular proteins studied by organ system (14, 15) as a guide to development of multiplexed SRM assays for targeted proteomics in the broader community.

In a matrix structure (Fig. 1), we established Resource Pillars of Mass Spectrometry led by Bruno Domon (later Yingming Zhao, Rob Moritz, and Susan Weintraub), Antibody Profiling led by Mathias Uhlen and Michael Snyder (later Emma Lundberg, Jochen Schwenk, and Cecilia Lindskog), Bioinformatics/Knowledgebase led by Amos Bairoch, Lydie Lane, and Eric Deutsch, and recently Pathology led by Daniel Chan, Edouard Nice, and Michael Roehrl. The MS pillar worked closely with the instrument companies and the Industrial Advisory Board. They conducted a needs survey and mounted an effort to stimulate a product line focused on high-throughput, moderate-cost instruments for clinical and epidemiological applications. However, the commitment to rapid progress on high-end, high-sensitivity instruments has carried the day; there is still a need for high-throughput instruments. The Antibody Profiling pillar gave visibility to arrays and aptamers, but became synonymous with the prodigious HPA (10, 16). The HPA has generated 31,000 antibodies directed at 18,000 proteins for immunohistochemistry and immunofluorescence of numerous tissues, cells, and organelles, now populating, together with transcriptomics, its Tissue, Cell, Pathology, Brain, Metabolism, Blood, and Secretome Atlases (17). The KB built upon the HUPO Protein Standards Initiative, the PRIDE database and ProteomeXchange at EBI, the new human-focused neXtProt resource associated with UniProtKB/SwissProt at the Swiss Institute of Bioinformatics in Geneva, and PeptideAtlas at the Institute for Systems Biology in Seattle. The Pathology pillar is dedicated to translation of proteomics and systems medicine to clinical applications in diagnosis and therapeutics.

Another significant feature of the HPP was the leading-edge experience and guidance of the Scientific Advisory Board of Michael Snyder (chair), Catherine Costello, Kun-liang Guan, Denis Hochstrasser, Leroy Hood, Matthias Mann, Kate Rosenbloom, Naoyuki Taniguchi, Mathias Uhlen, and John Yates; in 2020 Ruedi Aebersold became chair, with Subhra Chakraborty, Anne-Claude Gingras, Fuchu He, Kathryn Lilley, Emma Lundberg, Anthony Purcell, and John Yates. I had the privilege of chairing the HPP from 2010, followed by Mark Baker in 2018 and Rob Moritz in 2020.

## Data sharing and data quality

We were aware that the HGP had proposed and required prompt upload (within 24 h) of all sequence data from funded or contributing investigators. This feature led to concerns about problems with data quality, but the “fresh air of open disclosure” and advances in methodology progressively improved those submissions and their usefulness throughout the community. Without a funding lever, the HPP had only the power of persuasion and good examples, combined with emerging guidelines from the leading journals. *MCP* played a key role with the 2004 Carr *et al* paper on Publication Guidelines for Peptide and Protein Identification Data (18). These detailed guidelines addressed the diversity of mass spectrometers with embedded proprietary search engines and the need for transparency in generating tryptic peptides, evaluating mass spectra from peptide fragmentation, deducing peptide sequences, choosing reference genome sequences, and matching peptides to reference protein sequences in order to have any hope of replicability of results. Often the peptides matched to several or many protein sequences. The *MCP* guidelines mandated disclosure but not uniformity.

Data sharing required increasingly large-scale repositories for data sets and metadata. Early on it was recognized that fully informative proteomics data sets were much more complex than DNA sequence databases. Early resources included the Proteomics Identification Database (PRIDE) (19), Global Protein Machine DB (20), and Tranche Network (21). The Institute for Systems Biology created PeptideProphet/ProteinProphet and then the TransProteomicPipeline (TPP) (22) to address the pervasive problems of false-positive and ambiguous identifications. TPP facilitated uniform reanalysis of available data sets, downloaded from PRIDE or ProteomeCommons, or submitted directly to PeptideAtlas, to create PeptideAtlas builds. TPP introduced a requirement of peptide length ≥ 7 aa and later analyzed for contaminants using the Common Repository of Adventitious Proteins http://www.thegpm.org/cRAP.

**Fig. 2:**
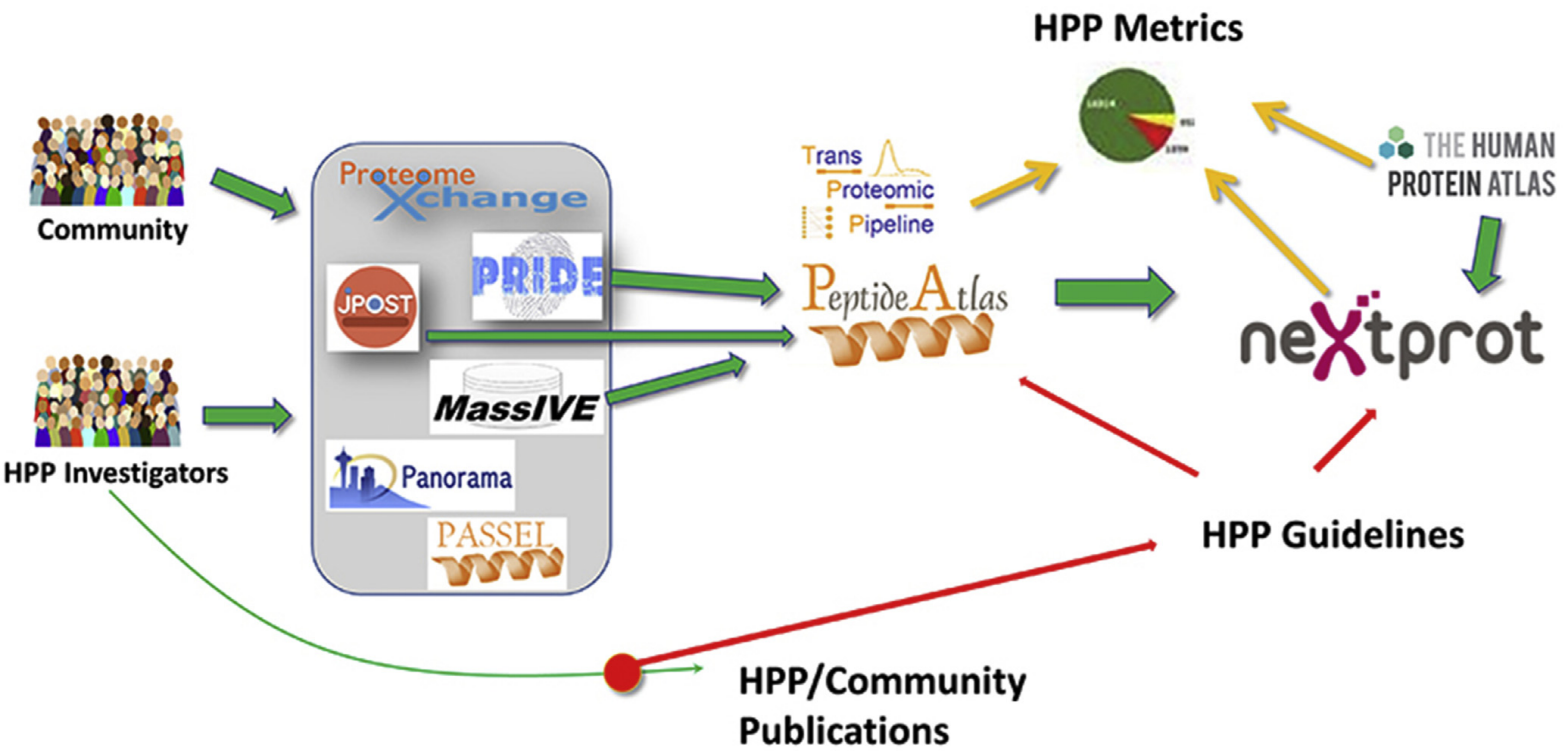
**The data flow for the Human Proteome Project, including the connectedness of ProteomeXchange with the major proteomics data set resources PRIDE and PeptideAtlas (founding partners), iProX, jPOST, MassIVE, and Panorama (modified from**www.proteomeXchange.org**)**.

From the 2008 US HUPO meeting, the HUPO 2008 Amsterdam Principles, and the 2010 Sydney International Cancer Proteomics Workshop, there were strong recommendations for a system for registration of data and metadata with open access; this became ProteomeXchange, based at EBI (19). The HPP in 2012 issued initial Guidelines for Interpretation of MS Data calling for registration of data sets in ProteomeXchange, open access to the data and metadata in Consortium resources, and use of a false discovery rate (FDR) of ≤0.01 at the protein level (not just peptide level). ProteomeXchange now connects data set submissions to multiple resources around the world (23) (Fig. 2).

Provided by Eric Deutsch.

The HPP recognized that claims of detection of protein expression in biological specimens used a wide variety of criteria for identification of specific proteins or “protein groups”. As illustrated in detail for the plasma proteome, PeptideAtlas contracted the 3020-protein human plasma proteome of 2005 (4), based on two peptide matches, to 2738 in the 2007 Build and then 1929 in 2010 (24). Remarkably, by 2016, 3509 plasma proteins (25) met the much more stringent HPP Guidelines for Mass Spectrometry Data Interpretation v2.1 (26).

neXtProt was created in 2010 at the Swiss Institute of Bioinformatics, based on UniProtKB and SwissProt, which had been operating since 1986. neXtProt was announced as the knowledge platform for the HPP in 2011 (27). neXtProt draws upon the curation processes and evidence levels of UniProtKB and SwissProt; it consolidates many resources with molecular data from studies of human specimens, including extensive sequence, splice isoform, and PTM information in the PEFF format from the HUPO Protein Standards Initiative. A “gold” level designation was considered to represent an error rate of ≤1%. neXtProt has depended on PeptideAtlas for mass spectrometry findings from its standardized reanalysis, with the addition of reanalyzed data sets from MassIVE in the 2020-01 (Jan) release, as described in the 2020 HPP JPR Metrics paper (28).

## Progress in Credibly Identifying the Protein Parts List and Reducing the Number of “Missing Proteins”: The Critical Role of HPP Guidelines

Here we address the first goal of the HPP, establishing the “parts list”. Each year the HPP has published a report on progress made throughout the global community toward credibly identifying and characterizing the complete protein parts list, as captured in neXtProt. Table 1 shows the evidence levels PE1 for high-quality protein-level evidence, PE2 for transcript evidence without adequate protein evidence, PE3 for protein homologs in nonhuman species, and PE4 for proteins predicted only from a gene model. PE2+PE3+PE4 represent the “missing proteins” (27). neXtProt also has a PE5 category (for dubious or uncertain protein-coding genes, including a high proportion of pseudogenes), which the HPP excluded from our missing protein effort in 2013. It is very important to recognize that the reference genomes (Ensembl, RefSeq, and others) and the UniProtKB, SwissProt, and neXtProt databases are changing dynamically each year as reference genomes are updated, new literature reports are evaluated and incorporated, and policy decisions are introduced (28).

**Table 1:** neXtProt protein existence evidence levels in releases from 2012-02 to 2020-01 showing progress in reducing the PE2,3,4 Missing Proteins, identifying proteins as PE1,^a^ and approaching a complete protein parts list (adapted from Omenn *et al* (28), JPR, 2020 and informed by Adhikari *et al* (12))

Level/date of neXtProt release	2012–02	2013–09	2014–10	2016–01	2017–01	2018–01	2019–01	2020–01
PE1: Evidence at protein level	13,975	15,646	16,491	16,518	17,008	17,470	17,694	17,874
Missing Proteins (MP) = PE2 + PE3 + PE4^b^	5511	3844	2948	2949	2579	2186	2129	1899
PE2: Evidence at transcript level	5205	3570	2647	2290	1939	1660	1548	1596
PE3: Inferred from homology	218	187	214	565	563	452	510	253
PE4: Predicted	88	87	87	94	77	74	71	50

PE1 = high-quality evidence for expression of the protein in compliance with HPP Guidelines; PE2 = detection of corresponding transcript without sufficient evidence of protein expression; PE3 = evidence of protein in nonhuman species; PE4 = protein predicted from a gene model, all according to neXtProt.

a PE1/PE1+2 + 3 + 4 = 17,874/19,773 = 90.4%.

b PE 2 + 3 + 4 = 1899 “missing proteins” as of neXtProt 2020-01 (Jan).

Entering 2012, we had a baseline of 13,975 PE1 proteins and a total of 5511 PE2,3,4 entries from neXtProt release 2012-02 (Feb) (11, 29). The initial HPP/PeptideAtlas Guidelines required two peptides of ≥7 aa in length and a FDR of 1% at the protein level (not just at the peptide level). We put a spotlight on the 5511 missing proteins and set out to find evidence of their expression with more extensive fractionation, higher mass accuracy MS, and more varied organ, tissue, and cell line specimens. We explicitly sought stronger evidence for PE1 proteins, as early studies counted multiple matches of peptides, short peptides, and proteins with only a single matching peptide (“one-hit wonders”). The early Plasma Proteome Project collaboration reported results in 2005 with alternative filters for number of peptides (4).

The TransProteomicPipeline of PeptideAtlas put a premium on controlling the FDR statistically at ≤1% for the protein level, which required much lower FDR at the peptide and PSM levels. If 10,000 protein matches were reported, FDR at 1%, let alone 5%, would mean that 100, or 500, proteins would be false-positives. Of course, they were not specifically identified, but it seemed likely that false-positives were among the lower abundance/previously undetected proteins being reviewed. When two large studies of multiple adult and fetal tissues were reported (30, 31) in 2014 with short peptides, single peptides, FDR <1% at the peptide level but no FDR limit at all at the protein level, and surprising claims of hundreds of olfactory receptors (ORs) being detected, a reassessment of guidance to the community was triggered. The HPP KB pillar developed Guidelines for MS Data Interpretation v2.1 (26), requiring clearly documented FDR ≤1% at the protein level for routine studies of known proteins. Studies making “extraordinary claims” of detection of previously missing proteins require a minimum of two uniquely-mapping/proteotypic, nonnested peptides of ≥9 aa in length whose mass spectra were carefully matched with spectra of synthesized peptides. These criteria represented the mantra from Amos Bairoch that “extraordinary claims require extraordinary evidence.” The Guidelines also directed investigators to rule out peptide matches to well-known, often abundant proteins with sequence variants or isobaric posttranslational modifications, both of which were frequent in the literature. neXtProt developed a uniqueness checker (SPARQL query NXQ-00022) to help investigators rule out matches to known proteins or single amino acid variants of known proteins. Meanwhile, Savitski *et al* (32) published a reanalysis of the two major papers that yielded much lower numbers of proteins in close keeping with the PeptideAtlas/HPP reanalyses (33). Ezkurdia *et al* (34) performed manual inspection of the mass spectra associated with the reported olfactory receptor proteins and were unable to confirm any of those claims. To this day, none of the 404 predicted OR proteins has been detected by mass spectrometry, even though *in silico* analyses predicted that semitryptic terminal peptides or missed cleavages could yield qualifying peptides (35); five ORs have been classified as PE1 in neXtProt from protein–protein interaction studies.

For the HPP metrics, the result of all of these quality enhancements from the HPP Guidelines v2.1 (26) was the demotion of 485 previously PE1 proteins in the neXtProt release of 2016-01, putting them into the PE2,3,4 missing proteins set (438 PE2, 40 PE3, and 7 PE4). As shown in Table 1, that substantially slowed the identification of PE1 proteins from 2014-10 to 2016-01. Separately, for 2016-01 there was a large increase in PE3 (from 214 to 565) due to a policy decision of UniProt/SwissProt to remove upgrades to PE2 that relied on inclusion in the ArrayExpress or CleanEx transcriptome repositories, greatly increasing the number of PE3 entries based on homology (detected in nonhuman species) (36). Later, the neXtProt 2020-01 release incorporated the merged RNA-seq data sets from Human Protein Atlas, GTEx, and FANTOM5 (37) to upgrade PE3 and PE4 entries to PE2 if the expression value were ≥1.0 RPKM (see Table 1, neXtProt Release 2020–01).

The most recent examples of a significant change in neXtProt due to a policy decision in UniProt/SwissProt are the consolidation of 71 PE1 HLA A,B,C genes and proteins into just seven entries, thus reducing the PE1 count by 64, and the addition of 19 entries of T cell receptors (TCR) (6 PE1, 13 PE4) (28).

## Celebrating a Major Milestone in Common with the Human Genome Project

### >90% of predicted proteins are now PE1 in neXtProt 2020-01

This invited Perspective complements HPP publications on “A High-Stringency Blueprint of the Human Proteome” and “The Human Proteome Reaches a Major Milestone” in special issues of *Nature Communications* (12) and the *Journal of Proteome Research* (28), respectively, released in conjunction with the 10th anniversary of the HUPO HPP. As documented in Table 1, there has been remarkable progress each year in gaining well-validated evidence for moving PE2,3,4 Missing Proteins to PE1, thereby reducing the number of PE2,3,4 entries. The ratio of PE1 to total PE1,2,3,4 proteins is now 17,874/19,733 = 90.4%. In analogy with the HGP, we celebrated this 90% milestone at the virtual HUPO Congress on October 19, 2020. In 2000, President Bill Clinton and Prime Minister Tony Blair staged a major event around progress in the HGP(s), “approaching 90% of the sequence” (38); in 2001, when the *Science* and *Nature* special issues were published, the leaders of the NIH and private sector initiatives declared 90% of the sequence established. Even today, there are major areas of repeated sequences and other anomalies to be sorted out in the human genome.

Though we have been discussing the likelihood of saturating the discovery of PE2, 3, or 4 proteins for several years, there appears to be surprisingly little evidence yet of saturating these two reciprocal curves (12, 28). There were 255 PE2,3,4 MPs converted to PE1 in the most recent year, from neXtProt 2019-01 to 2020-01. Of the 17,874 PE1 proteins, 16,924 are based on validated MS results, of which 16,655 represent canonical proteins in the 2020-01 PeptideAtlas build. The large MassiVE data repository (39) was utilized for the first time in updating neXtProt, adding 84 proteins found only in MassIVE to the 16,836 found in both or only in PeptideAtlas (28).

Among many notable developments from the focus on missing proteins by the chromosome-centric arm of the HPP, I here highlight these five: (a) Chromosome 2 and 14 teams in Switzerland and France and Chr 1 in China applied the HPA evidence of nearly exclusive expression of >800 transcripts in testis (40) to multiyear analyses of sperm, testis, and other male reproductive specimens, with outstanding results (41, 42). The Sun *et al* (42) data set yielded 73 new canonical proteins at PeptideAtlas, then PE1 proteins at neXtProt. There is more gold to be mined in the testis. (b) A study of sumoylation represented a dramatic example of enrichment for PTMs, leading to 269 previously undetected proteins (43); another enrichment technique used ProteoMiner beads for adsorption of similar amounts of all proteins, with washing away the excess of higher abundance proteins (44). (c) The special resource created by the B/D-HPP and the MS pillar, the SRM Atlas (13), was applied to *in silico* matching of spectra from pairs of peptides meeting HPP Guidelines, captured in the same study in GPMdb, and recovered from PRIDE (45). We have learned that few investigators with multiple missing protein candidates prepare pairs of synthetic peptides for all of their MP candidates, so the use of SRM Atlas, now combined with the Universal Spectrum Identifier [http://psidev.info/USI] (46), is quite helpful. (d) Membrane proteins are notoriously difficult to solubilize and generate tryptic peptides due to high hydrophobicity; nevertheless, the data sets from Zhang *et al* (47) and Weldemariam *et al* (48) provided 48 and 40 additional PE1 proteins, respectively, *via* PeptideAtlas. (e) A major phenomenon from MS is the confirmation of expression of many PE1 proteins that were classified by SwissProt curators based on non-MS protein evidence in the published literature that are now classified based on MS evidence as meeting the stringent HPP MS Guidelines 2.1 and 3.0 (46). In 2016, there were 1860 PE1 proteins based on such non-MS evidence; as of neXtProt release 2020-01, that number has been reduced to 950. Of these, 73 are based on Edman sequencing, 122 on disease mutations, 35 from 3D structures, 342 from protein–protein interactions, 49 from antibody-based techniques, 127 from PTMs and processing, and 202 from biochemical studies. Many have multiple lines of evidence; these numbers represent the first type of evidence curated for each protein. We have taken note that there are currently no formal guidelines for these types of studies. We have initiated a review of the evidence for the PE1 proteins based on protein–protein interactions.

## Why Are 1899 Predicted Proteins Still Unidentified? How May They Be Detected?

It is feasible to examine the reasons why each PE2, PE3, or PE4 protein is still missing protein-level evidence and plan a specific strategy for finding it. Such an analysis was performed by the Chr 17 team as part of the C-HPP Next-50 MP Challenge of October 2016 for each chromosome team. A combination of MS and protein–protein interaction studies had yielded 40 of the first 43 MPs detected (49) (now there are 18 more (28)). For the remaining 105 MPs, the prospects for detection by MS were examined: 89 had at least two predicted proteotypic tryptic peptide sequences; 27 of those already had one uniquely mapping peptide in PeptideAtlas; and 61 had well-expressed transcripts in specific tissue types, including 24 in testis and 8 in cerebellum. Among families of TBC1D and of keratin-associated proteins, which occur in large clusters, the sequence homology is so high as to make differentiation difficult by MS, but potentially feasible by PPI (49).

For many MPs, transcript levels may be undetectable or very low; Sjostedt *et al* (37) estimated that 800–1000 genes had no transcript expression >1.0 RPKM in any tissue studied by Human Protein Atlas, FANTOM5, or GTEx, including 399 olfactory receptors (12) and 32 of 36 beta-defensins (which may be expressed only in response to infection or inflammation). Use of multiple proteases, which do generate more peptides, has yielded only a few additional proteins so far in multiple studies; they may need to be applied in combination with solubilization and deep fractionation to gain sensitivity. N- and C-terminal peptides and peptides with missed cleavages can be useful, as they account for some proteins lacking tryptic sites that are already PE1 by MS.

There is little doubt that the major challenge in detecting MPs is low abundance of the protein in all tissues studied, regardless of transcript levels. Enrichment should be a productive strategy, targeting PTMs or using adsorptive beads, as cited above. Enrichment with specific antibodies has long been recommended; a collaboration between the HPA and the Chr 14 team for studies of the many testis-specific MPs remaining to be detected is underway. Meanwhile, mass spectrometers continue to gain sensitivity and mass accuracy. Yet another major source of MPs is high homology among members of protein families, resulting from duplicated genes; finding two uniquely mapping peptides of ≥9 aa length may be difficult. Currently, each protein must be confidently detected and distinguished to be counted; in earlier times one or two or many of a “protein group” would have been counted without knowing exactly which gene product had been measured. Unusual tissues and cell types remain understudied. Given the remarkable cellular and circuitry heterogeneity of brain regions and the HPA evidence for 318 brain-specific transcripts, more in-depth analyses of subregions and pathways in the brain should be especially productive.

## Emphasizing Functional Annotation of neXtProt Proteins

A major commitment of the protein parts list approach is to characterize the functions and properties of the proteins and their splice variants and PTM proteoforms. Of the now 17,874 PE1 proteins, 1254 lack annotation for function using specific Gene Ontology terms. In fact, it has often been noted that about 90% of protein studies focus on the 10% most studied proteins, suggesting that there is much to be learned about less-studied proteins. In 2017, the C-HPP launched the CP50 Initiative to stimulate experimental studies in support of functional annotation and characterization of these uPE1 proteins (50). Elaborate studies have been conducted to characterize individual proteins (51). In parallel, a computational approach utilizing I-TASSER/COFACTOR algorithms for protein folding and protein function prediction is now available upon request *via* the neXtProt community button on protein-specific pages (52). Together the missing proteins and unannotated proteins constitute “the dark proteome” (50).

## The Biology and Disease-driven HPP: Creating an Emphasis on Proteogenomics

The 19 teams of the biology and disease-driven HPP are identified in Figure 1 [https://hupo.org/B/D-HPP]. The second HPP goal of integrating proteomics into all multiomics research has been frustrating, though there is notable progress. Spatiotemporal quantitative analyses of protein expression, pathways, and networks have been a major theme of the B/D-HPP (53). In 2011, a Working Group strongly recommended a cross-agency US Life Sciences Grand Challenge on Proteomics Technologies (54). The EU-funded Proteomics Specifications in Time and Space (PROSPECTS) Network reported in a special issue of MCP in 2012 significant progress toward revolutionizing cell biology (55). The Network brought together improved resolution and sensitivity of the Orbitrap family of instruments, antibody applications, and quantitation of protein dynamics. In 2014, Nesvizhskii (56) proposed proteogenomics concepts and guidelines for customized protein sequence databases generated using genomic and transcriptomic information to help identify novel peptides not present in reference protein sequence databases from mass-spectrometry-based proteomic data. Conversely, protein findings can guide refinements in the reference genomes.

Our largest B/D collaboration, for cancers, is with the National Cancer Institute’s Clinical Proteomic Tumor Analysis Consortium (CPTAC), which has developed resources for integrated omics analyses of multiple cancer types. Building upon the TCGA consortium of a decade earlier, which had only a small RPPA reverse array proteomics component, CPTAC3 combines copy number variation, whole genome and whole exome sequencing, DNA methylation, RNA-seq, miRNAs, global proteome, phosphoproteome, sometimes acetylome and ubiquitinome, and immune subtyping for a rapidly growing series of specific cancers [https://cptac-data-portal.georgetown.edu/cptacPublic/]. The global proteome and phosphoproteome analyses, especially of kinases and their substrates, identify novel biological features and likely targets for precise therapeutic interventions with chemotherapy or immune therapies. The most recent examples are subtypes of clear cell Renal Cell Adenocarcinomas (57) and Lung Adenocarcinomas (58). The Liver Proteome team in China has long focused on hepatocellular carcinomas (HCC). Their most recent work identified in early hepatocellular cancers due to chronic hepatitis B virus infection three subtypes, including one with a striking target, sterol O-acetyl transferase (SOAT1), shown to be responsive to therapy in patient-derived-explant (PDX) models in mice (59).

Another major multiomics project with ties to the HPP is the NIH Common Fund Molecular Transducers of Physical Activity Consortium (MoTrPAC). Preclinical and clinical studies examine the systemic effects of endurance and resistance exercise and fitness levels in children from age 10 and adults by molecular probing especially of skeletal muscle and adipose tissues. Proteomics methods include untargeted MS with TMT-labeling and targeted aptamer assays. MoTrPAC’s public database is expected to enhance understanding of the health benefits of exercise and provide insight into how physical activity mitigates disease (60). A separate skeletal muscle proteomics study identified age-associated changes in alternative splicing and autophagy connected to decline in skeletal muscle function (61).

The B/D Infectious Disease team has noted widespread application of MALDI-Tof-MS in clinical microbiology (12). MALDI-MS detects diverse molecules, including lipid A, glycans, and proteoglycans. Viral infections represent outstanding opportunities for basic and clinical studies involving evolution of viral invasion strategies and host defenses, *e.g.,* protein acetylation in human cytomegalovirus infection (62). Posttranslational modifications have proved valuable guides to biomarker discovery and application also in cardiovascular disorders (63).

## Commitment to Career Development of Early Career Researchers

Led by B/D-HPP chair Jennifer van Eyk, the HPP in mid-decade mobilized several featured programs for young investigators, including a Mentoring Day at each World Congress, a manuscript competition, poster sessions, opportunities to serve as comoderators for panels, and engagement in the HPP day-long strategy workshops. Maggie Lam in the van Eyk/Ping group (14) and KH Yu in the Snyder group (15) utilized advanced bibliometric techniques to identify “popular” or “priority” proteins, respectively, from the published literature around each organ or disease and highlight proteins that could be incorporated into multiplex targeted assays for widespread utilization; several B/D teams are utilizing these protein sets in combination with SRM targeted proteomics or DIA-SWATH.

## Directions for the Human Proteome Project for the Coming Decade

(1) There is much more to be done pursuing the two long-standing goals of the HPP: (a) the identification and characterization of protein products and their proteoforms from each protein-coding gene, and (b) the establishment of proteomics as an integral component of all kinds of multiomics research critical to understanding basic biology, pathophysiology, and precision health/precision medicine. This work has progressed well and will surely accelerate (64). The HPP Guidelines will continue to enhance the quality and replicability of research in this broad domain.
(2) There will be much deeper and more quantitative analyses of networks, pathways, and systems with the addition of machine learning, artificial intelligence, and deep learning to bridge the fields and enhance the feasibility of analysis of very large data sets. An example of synergy is the bridge between structural biology of proteins, including uses of cryo-EM, and proteomics. An early application was the report of computational predictions of conformation and folding for pairs of splice isoforms (65).
(3) Methods are emerging for protein analyses at the level of single cells, a burgeoning area of integration with RNA-Seq, using mass cytometry, live-cell imaging, and computational tools (66). Understanding the heterogeneity of organ and tissue function at the cellular level during differentiation and in response to therapy is already a central theme in oncology and is likely to become very important for the brain, kidney, liver, lung, and other organs.
(4) There is rapidly growing attention to the vast domain of the genome outside the 1.2% coding for proteins. Early interest in smORFs and lncRNAs having translation products foundered on the lack of convincing spectral evidence for the identification of such proteins or polypeptides (36). Now, ATAC-Seq can detect tissue-specific enhancers and super-enhancers from the nonprotein-coding DNA (67, 68). Hi-C cross-linking analyses identify functionally associated proteins from coexpressed genes and formation of transcription-associated domains (TADs) and transcriptional condensates (69, 70). The Encyclopedia of DNA Elements (ENCODE) phase III has expanded analysis of the cell and tissue repertoires of transcripts, chromatin structure and modification, DNA methylation, chromatin looping, and occupancy by transcription factors and RNA-binding proteins (71). It is timely for proteomics to add functional depth to the biological interpretation of those methods.
(5) The long-gestating promise of protein biomarkers for early diagnosis of clinical disorders warrants renewed effort that takes into account interactions of proteins in macromolecular complexes, biological effects of splice isoforms, and dynamic effects of proteins with a wide variety of posttranslational modifications (72). Such studies are emerging from the CPTAC3 Consortium and from the CVD-B/D HPP.
(6) High throughput, cost-effective assays for proteomic biomarkers are emerging with very rapid, sensitive, and robust fractionation/MS analysis using Evosep One (73, 74). Affinity-based methods such as O-Link (75) and SOMAScan (76) are also being deployed. It is essential to cross-validate findings with these different platforms and to establish a role for proteomics methods in the very large-scale population studies such as UK Biobank and AllofUs.
(7) Finally, I hope that complementary proteomics methods will be applied for orthogonal confirmation and validation of findings, as is being planned for mass spectrometry and immunohistochemistry on testis and other organs, and for the neXtProt PE1 entries not yet based on mass spectrometry, to enhance confidence in the proteomic findings and to more fully characterize the proteome in systems biology context, thereby preparing for their application to precision medicine and precision health.

## Conflict of interest

The author declares no competing interests.
